# Prevalence of Metabolic Syndrome in Patients With Rheumatoid Arthritis: An Updated Systematic Review and Meta-Analysis

**DOI:** 10.3389/fmed.2022.855141

**Published:** 2022-04-08

**Authors:** Wei Cai, Xuemi Tang, Min Pang

**Affiliations:** ^1^Pediatric Department, Huazhong University of Science and Technology Union Shenzhen Hospital, Shenzhen, China; ^2^Department of Rheumatology and Immunology, Children's Hospital of Chongqing Medical University, Chongqing, China

**Keywords:** metabolic syndrome, rheumatoid arthritis, prevalence, systematic review, meta-analysis

## Abstract

**Introduction:**

Rheumatoid arthritis (RA) due to systemic inflammation and insulin resistance increases the risk of cardiovascular disease and reduces life expectancy. In order to develop cardiac death prevention strategies, it is necessary to estimate the prevalence of metabolic syndrome (MetS) in these patients.

**Methods:**

This systematic review and meta-analysis was performed to estimate the prevalence of MetS among patients with RA. International databases (i.e., Scopus, PubMed, Web of Science, and Google Scholar) were searched during the period of October 1 and October 10, 20121. Heterogeneity among the included studies was assessed through the Cochrane Q test statistics and I^2^ test. Finally, a random-effects meta-analysis model was computed to estimate the pooled prevalence of MetS.

**Results:**

Sixty-one articles with 96 groups and a sample size of 13,644 people were analyzed. The pooled prevalence of MetS was 32% (95% CI: 29.6–34.4). The highest prevalence of MetS is related to studies conducted in Asia (32.7%, 95% CI: 29–36.3) and Europe (32.7%, 95% CI: 27.5.37.9) and the lowest Prevalence was also related to studies conducted in Africa (28%, 95% CI: 28.8–32.2). The prevalence of MetS in men was 33% (95% CI: 26–39) and 34% (95% CI: 29–40) in women. Findings by diagnostic criteria showed that the highest and lowest prevalence of MetS was related to ATP III (37.5%, 95% CI: 30.9–44.2) and EGIR (14.4%, 95% CI: 10.5–18.5), respectively.

**Conclusions:**

MetS is highly prevalent in patients with RA and identification of high-risk patients is necessary to prevent cardiovascular mortality.

## Introduction

Rheumatoid arthritis is a chronic inflammatory disease of unknown etiology characterized by systemic symptoms, especially joint involvement and deformity (1). Patients with rheumatoid arthritis are at high risk for cardiovascular disease and premature death due to systemic inflammation, which reduces their life expectancy by 5 to 10 years (2, 3). Rheumatoid arthritis is associated with insulin resistance, dyslipidemia, and changes in adipokines profiles that are components of the metabolic syndrome (MetS) (4). Insulin resistance is a constant risk factor for cardiovascular disease and the central mechanism in metabolic syndrome, which is present in 70% of patients with RA (5, 6).

MetS, also known as syndrome X and insulin resistance syndrome, refers to a set of cardiovascular risk factors (obesity, glucose intolerance, dyslipidemia, and high blood pressure) that can lead to cardiovascular disease (7). MetS increases cardiovascular outcomes and mortality by 2 and 1.5 times, respectively (8, 9). The increased risk of cardiovascular disease in patients with rheumatoid arthritis has been well established, so that the European League Against Rheumatism (EULAR) recommends that screening and management of cardiovascular risk in these patients be performed immediately (10, 11).

Various studies have shown that the prevalence of metabolic syndrome in these patients varies between 10 and 56% (12, 13). In this systematic review and meta-analysis, the cumulative prevalence of metabolic syndrome in patients with rheumatoid arthritis has been estimated.

## Methods

### Search Strategy

The present systematic review and meta-analysis study was performed according to Preferred Reporting Items for Systematic Reviews and Meta-Analyses (PRISMA) guidelines (14). To access articles examining the prevalence of metabolic syndrome in patients with rheumatoid arthritis, a comprehensive search with no data limit was performed in the following databases: PubMed/MEDLINE, Scopus, Web of Science, and Google Scholar. The search was conducted between October 1 and October 10, 2021. All article published until August 30, 2021 were included. Articles were searched with keywords (“Metabolic Syndrome”[Mesh] OR “Metabolic Syndrome^*^”[tiab] OR “Insulin Resistance Syndrome^*^”[tiab] OR “Metabolic X Syndrome^*^”[tiab] OR “Dysmetabolic Syndrome^*^”[tiab] OR “Reaven Syndrome^*^”[tiab] OR “Metabolic Cardiovascular Syndrome^*^”[tiab]) AND (“rheumatic diseases”[Mesh] OR “Arthritis, Rheumatoid”[Mesh] OR “Rheumatic disease^*^”[tiab] OR “Rheumatism^*^”[tiab] OR “Rheumatoid Arthritis”[tiab] OR “Rheumatic symptom^*^”[tiab]) AND (“Prevalence”[Mesh] OR “Prevalence^*^”[tiab] OR “Period Prevalence^*^”[tiab] OR “Point Prevalence^*^”[tiab]). The reference lists of the included articles were also reviewed to find other eligible articles.

### Selection of Studies and Data Extraction

All observational studies published in English that reported the prevalence or frequency of metabolic syndrome in patients with rheumatoid arthritis were analyzed. Interventional, review, and replication studies, as well as studies investigating the prevalence of metabolic syndrome in other rheumatic diseases, were excluded. According to the inclusion and exclusion criteria, the titles and abstracts of the articles were independently reviewed by two researchers and the required information such as first author, year of publication, country of study, sample size, prevalence or frequency of metabolic syndrome in patients with rheumatoid arthritis were extracted and recorded in a pre-prepared form. To evaluate the quality of articles, the modified Newcastle-Ottawa Scale (NOS) was used, which has three main sections. The first part, rated on a scale of one to five stars, focuses on the methodological quality of each study (i.e., sample size, response rate, and sampling technique). The second section considers the comparability of the study cases or cohorts with a possibility of two stars to be gained. The last section is concerned with the outcomes and statistical analysis of the original study with a possibility of three stars to be gained. Two authors extracted the information and evaluated the methodological quality of the articles, independently. Any disagreements between the two reviewers were resolved consensus (15, 16).

### Statistical Analysis

Point estimation and 95% confidence interval (CI) of metabolic syndrome due to binomial distribution formula and heterogeneity between studies was evaluated by Cochran Q test with a significance level of less than 0.1 and I^2^ index. The degree of heterogeneity was assessed using the I^2^ index. Heterogeneities were divided into three categories: less than 25% (low heterogeneity), 25 to 75% (moderate heterogeneity) and more than 75% (high heterogeneity). Pooled prevalence was estimated using a random-effects model. Subgroup analysis was performed based on diagnostic criteria and continent. To investigate the potential publication bias, funnel plot based on Egger's regression test was used. Univariate meta-regression was used to investigate the relationship between the prevalence of metabolic syndrome and the year of study and the mean age of patients. Data analysis was performed using Stata software version 16.

## Results

**Figure 1:**
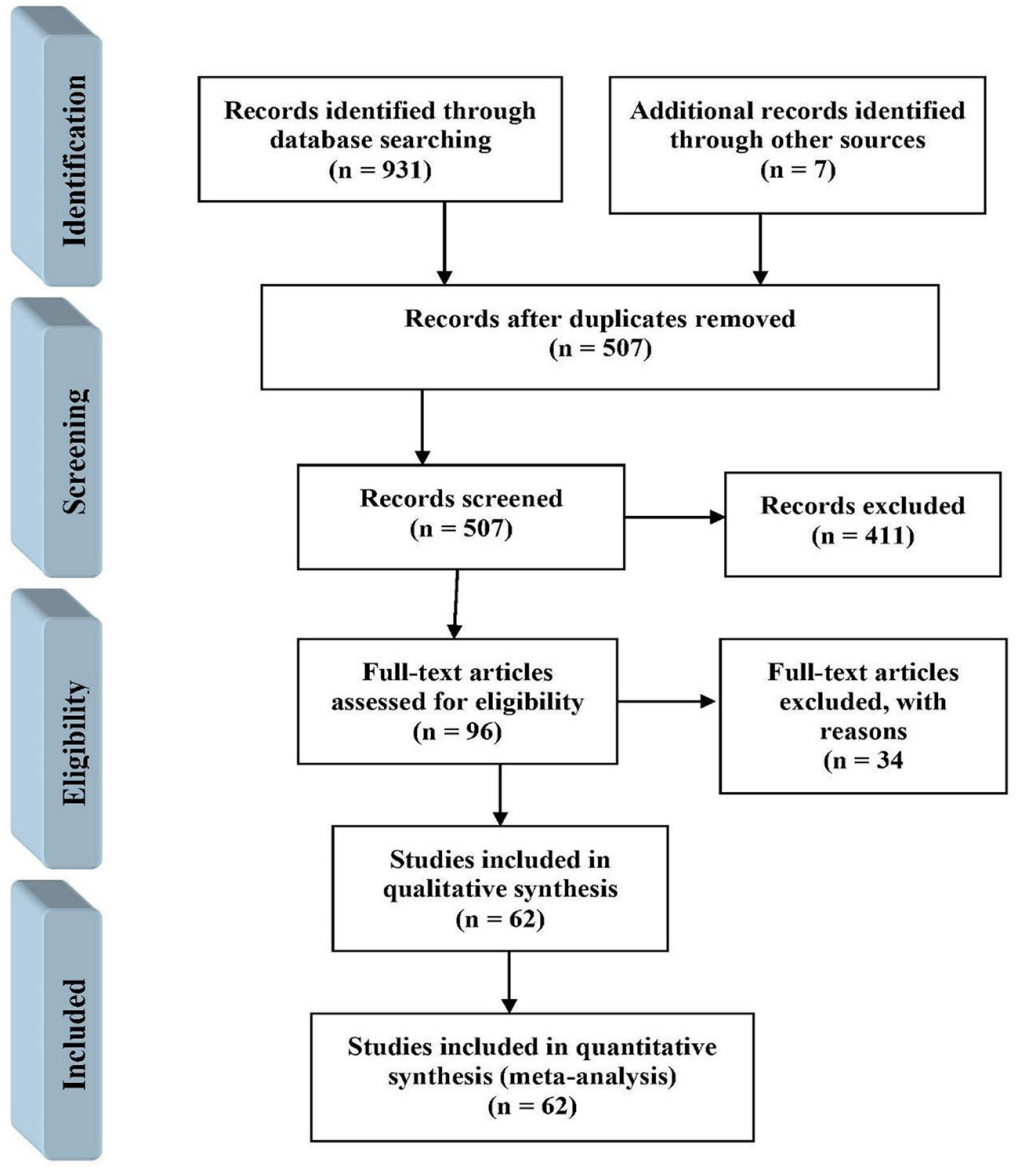
Study selection following PRISMA guidelines.

In the initial search, 938 potentially relevant articles were retrieved. Of these articles, 431 articles were excluded due to duplications and removing duplicate articles, 507 articles remained. The titles and abstracts of the remaining articles were reviewed and 411 irrelevant articles were removed. Of the remaining 96 articles, 34 articles were deleted for not reporting the prevalence of MetS (Figure 1).

### Study Characteristics

In this study, 62 articles with a sample size of 13,644 people were analyzed, the characteristics of which are listed in Table 1. Most studies were performed in Morocco (*n* = 9) and Iran (*n* = 9). Most studies were based on NCEP/ATP III (*n* = 42) and IDF (*n* = 21) diagnostic criteria. Thirty-nine studies were conducted in Asia, 25 in Europe, 18 in the United States and 14 in Africa. All selected articles had good methodological quality.

**Table 1:** Characteristics of included articles.

**First author**	**Year**	**Country**	**Sample size**		**Diagnostic criteria**	**Mean age**	**RA patients (%)**		
			**Total**	**M/F**			**Total**	**Male**	**Female**
Turgunova et al. (17)	2021	Kazakhstan	101	31/70	IDF	-	40.5	-	-
Hee et al. (18)	2021	Singapore	561	0/561	NCEP/ATP III	-	44.9	-	-
					JC 2009	-	49.4	-	-
Giraud et al. (19)	2021	France	75	20/55	WHO	59.2	28	-	-
Kong et al. (20)	2021	China	717	152/565	CDS	61	31.2	-	-
Cioffi et al. (21)	2021	Italy	228	-	IDF	58	15	-	-
Mobini et al. (13)	2020	Iran	200	-	NCEP/ATP III	-	54.5	-	-
					IDF		56	-	-
Garcia-Chagollan et al. (4)	2020	Mexico	216	22/194	NCEP/ATP III	46	30.6	-	-
					ALAD		28.7	-	-
Xu et al. (22)	2020	Korea	247	48/199	NCEP/ATP III	58	15	-	-
Shaikh et al. (23)	2020	Pakistan	104	10/94	NCEP/ATP III	33.4	32.7	-	-
Ozkul et al. (24)	2019	Turkey	50	11/39	IDF	56.9	36	-	-
Mulumba et al. (3)	2019	Congo	75	15/60	NCEP/ATP III	51.8	25.3	-	-
Ene et al. (25)	2019	Romania	120	31/89	IDF-NCEP/ATP III	52.7	39.2	45.2	37.1
Naidu et al. (26)	2019	India	114	21/93	NCEP/ATP III	44.8	31.6	-	-
Kuriya et al. (27)	2019	USA	1543	443/1100	WHO	54	30.8	42	26
Akbal et al. (28)	2019	Turkey	53	12/41	ATP III	51	47.1	-	-
Aleksic et al. (29)	2019	Serbia	81	19/62	IDF	59.7	54.3	-	-
Mobini et al. (30)	2018	Iran	140	25/115	NCEP/ATP III	44.7	31.4	-	-
					IDF		35	-	-
Gomes et al. (7)	2018	Brazil	338	31/307	NCEP/ATP III	53.5	51.3	-	-
Burggraaf et al. (31)	2017	Netherland	212	65/147	NCEP/ATP III	54	40.1	-	-
Slimani et al. (32)	2017	Algeria	249	36/213	NCEP/ATP III	50.1	13.9	14.3	13.8
Pandey et al. (33)	2017	India	84	18/66	ATP III 2004	44.8	39.2	-	-
Ostojic et al. (34)	2016	Serbia	36	6/30	-	36	30.6	-	-
Lee et al. (35)	2016	Korea	598	110/488	AHA/NHLBI	63.6	36.4	34.5	36.9
Hugo et al. (36)	2016	France	57	15/42	IDF	57.6	24	25	24
Zafar et al. (37)	2016	Pakistan	384	97/277	NCEP/ATP III	43.8	31.3	18.5	35.5
Oliveira et al. (38)	2016	Brazil	107	0/107	NCEP/ATP III	55.5	51.4	-	51.4
					IDF		53.4	-	53.4
Muller et al. (39)	2016	Estonia	91	66/25	NCEP/ATP III	51.6	35	-	-
Dihingia et al. (40)	2016	India	72	6/66	NCEP/ATP III	41.5	16.7	-	-
Ghazaly et al. (41)	2015	Egypt	80	13/67	ATP III	40.7	50	53	49.2
Salamon et al. (42)	2015	Croatia	583	100/483	ATP III	59	43.1	40	43.7
Tantayakom et al. (43)	2015	Thailand	267	31/236	NCEP/ATP III	59	16.1	12.9	16.5
Parra-Salcedo et al. (44)	2015	Mexico	160	18/142	AHA/NHLBI	38.1	28	-	-
					IDF		18	-	-
					NCEP/ATP III		24	-	-
Craciun et al. (12)	2014	Romania	51	7/44	IDF-AHA	55.2	19	10.5	82.4
					NCEP/ATP III		23	-	-
					IDF		18	-	-
					AHA		14	-	-
Bilecik et al. (45)	2014	Turkey	100	0/100	IDF	52	33	-	33
					NCEP/ATP III		27	-	27
Ozmen et al. (46)	2014	Turkey	52	15/37	NCEP/ATP III	51	17.3	-	-
					WHO		28.8	-	-
Kumar et al. (47)	2014	India	54	6/48	IDF	46	29	-	-
					NCEP/ATP III		31	-	-
Abourazzak et al. (48)	2014	Morocco	179	22/157	IDF	49	30.7	-	-
					NCEP/ATP III		29	-	-
					AACE 2003		24	-	-
Salinas et al. (49)	2013	Argentina	409	69/340	ATP III	55.5	30	62	23.8
					IDF		35	-	-
Abdul-Qaharr et al. (50)	2013	Iraq	203	41/162	NCEP/ATP III	46.9	51.2	12	92
Rostom et al. (51)	2013	Morocco	120	10/110	NCEP/ATP III 2004	49	30.8	10	32.7
					NCEP/ATP III 2001		24.6	-	-
					WHO		20	-	-
					IDF		48.6	-	-
					EGIR		18	-	-
					JC 2009		32.3	-	-
Lee et al. (52)	2013	Korea	84	0/84	NCEP/ATP III	50.6	19	-	19
Ormseth et al. (53)	2013	USA	162	18/144	ATP III	54	26	-	-
Karakoc et al. (1)	2012	Turkey	54	7/47	IDF	49.8	42.6	-	-
Manka et al. (54)	2012	Slovakia	87	4/83	IDF	58.8	48.3	-	-
					NCEP/ATP III		44.8	-	-
					AHA/NHLBI		47.1	-	-
Da Cunha et al. (55)	2012	Brazil	283	50/233	NCEP/ATP III	56.8	39.2	-	-
Goshayeshi et al. (56)	2012	Iran	120	14/106	NCEP/ATP III	45.5	45.2	-	-
Baker et al. (57)	2012	USA	499	83/416	IDF	49.5	10.6	-	-
Crowson et al. (58)	2011	USA	232	58/174	NCEP/ATP III	58.8	33	36	32
Sahebari et al. (59)	2011	Iran	120	14/106	IDF	45.5	30.8	28.6	41.5
					NCEP/ATP III		45.2	28.6	37.7
Karimi et al. (60)	2011	Iran	92	0/92	NCEP	48.3	27.2	-	27.2
					WHO		19.6	-	19.6
Mok et al. (61)	2011	Hong Kong	699	133/566	JS 2009	53.3	20	-	-
Dao et al. (62)	2010	Vietnam	105	0/105	IDF	56.3	40.9	-	-
					NCEP/ATP III 2004		32.4	-	-
					NCEP/ATP III 2001		24.7	-	-
					JS 2009		32.4	-	-
					WHO		19	-	-
					EGIR		16.2	-	-
Raterman et al. (63)	2010	Netherland	236	79/157	NCEP	62.1	19.9	-	-
Solomon et al. (64)	2010	South Africa	291	32/259	NCEP/ATP III	27.2	31.3	-	-
			335	65/270	NCEP/ATP III	27.2	20.3	-	-
Giles et al. (65)	2010	USA	131	51/80	NCEP/ATP III	61	36	-	-
Santos et al. (66)	2010	Portugal	98	0/98	ATP III	49.2	25.5	-	-
Toms et al. (67)	2009	UK	387	105/282	IDF	63.1	45.3	52.7	42.6
					NCEP/ATP III 2004		40.1	42.5	39.2
					NCEP/ATP III 2001		38.3	40	37.7
					WHO		19.4	25.5	17.2
					EGIR		12.1	22.6	8.2
Chung et al. (2)	2008	USA	66	18/48	WHO	59	42	-	-
Zonana-Nacach et al. (68)	2008	Mexico	107	-	NCEP/ATP III	42.9	18.7	-	-
Karvounaris et al. (69)	2007	Greece	200	53/147	ATP III	63	44	39.6	45.6
Montagna et al. (70)	2007	Italy	45	3/42	NCEP/ATP III	53.8	55.5	-	-

**Figure 2:**
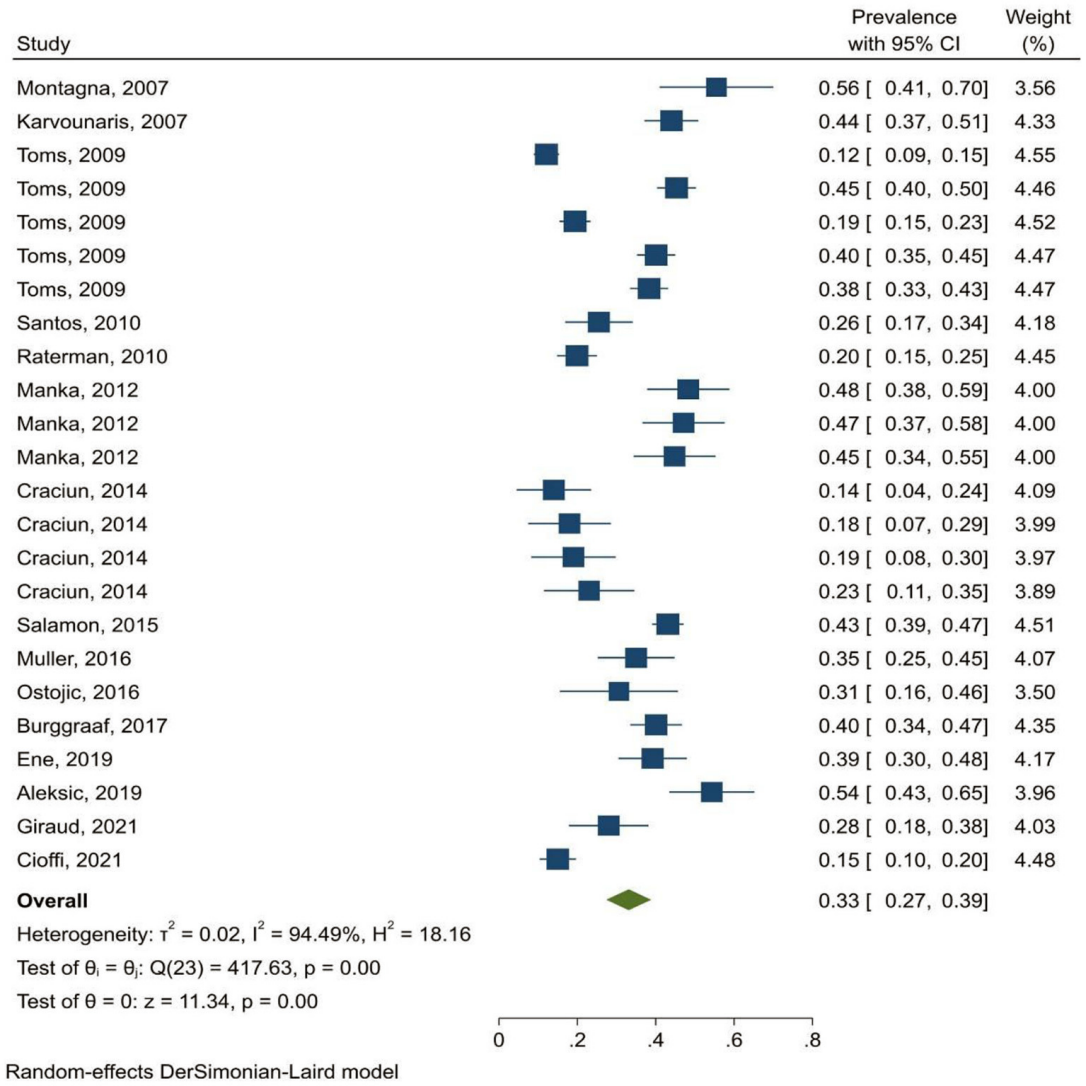
Forest plot of the pooled prevalence of MetS in patients with RA in Europe.

The prevalence of MetS in patients with rheumatoid arthritis was 32% (95% CI: 29.6–34.4%). The prevalence of metabolic syndrome was 33% (95% CI: 26–39%) in men and 34% (95% CI: 29–40%)in women. The findings demonstrated that the highest prevalence of MetS was related to studies in Asia (32.7%, 95% CI: 29–36.3%) and Europe (32.7%, 95% CI: 27.5–37.9%) and the lowest prevalence was related to studies in Africa (28%, 95% CI: 22.8–33.2%) (Figure 2). Findings by diagnostic criteria of metabolic syndrome showed that the highest and lowest prevalence were related to ATP III (37.5%, 95% CI: 30.9–44.2%) and EGIR (14.4%, 95% CI: 10.5–18.5%) criteria, respectively (Table 2).

**Table 2:** Subgroup prevalence of MetS among patients with RA.

**Subgroups**	**Number of studies**	**Prevalence (95% CI)**	**Between studies**			**Subgroup**	
			**P_**heterogeneity**_**	**Q**	**Q**	**P_**heterogeneity**_**	**I^**2**^**
**Continent**							
Asia	39	32.7 (29–36.3)	91.25%	0.001	505.13	2.39	0.495
Europe	25	32.7 (27.5–38)	93.37%	0.001	418.57		
America	18	32.3 (27–37.5)	94.66%	0.001	345.11		
Africa	14	28 (22.8–33.2)	88.24%	0.001	155.11		
**Criteria**							
WHO	8	25.2 (20–30.4)	81%	0.004	42.19	79.69	0.001
IDF	21	35.2 (29.4–41.1)	93.1%	0.017	482.13		
JS	4	33.5 (21–46)	95.6%	0.015	128.65		
NCEP/ATP III	42	32 (28.5–35.5)	91.2%	0.012	518.62		
ATP III	8	37.5 (31–44)	85.9%	0.007	47.09		
AACE	4	26.2 (17.3–35.2)	87.8%	0.007	25.17		
EGIR	3	14.4 (10.5–18.4)	36.75	0.001	2.92		

*WHO, World Health Organization; IDF, International Diabetes Federation; EGIR, European Group against Insulin Resistance; NCEP ATPIII, National Cholesterol Education Program Adult Treatment Panel; AACE, American Association of Clinical Endocrinologists; AHA/NHLBI, The American Heart Association / National Heart, Lung, and Blood Institute; JS, Joint Statement*.

### Meta-Regression

**Figure 3:**
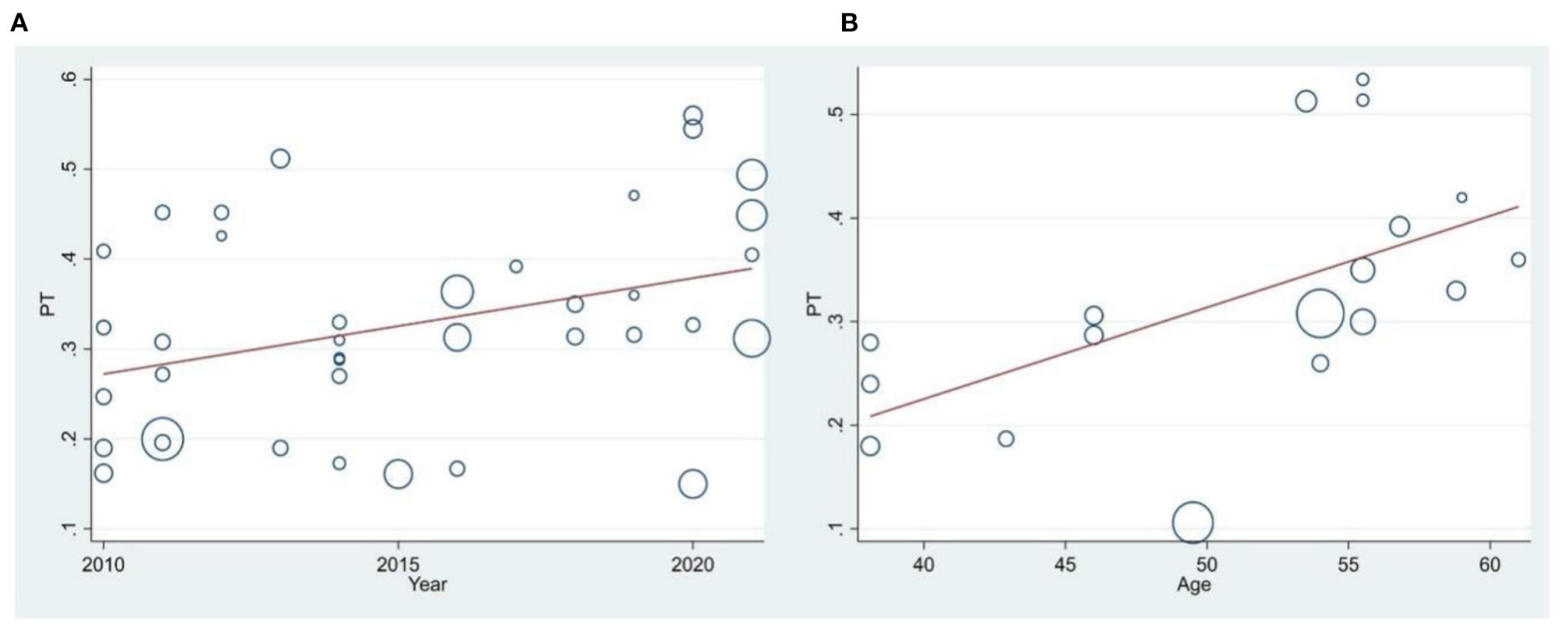
Meta-regression of the prevalence of MetS among patients with RA based on year of study **(A)** and mean age **(B)**. Circles indicate the weight of studies.

The results of meta-regression showed that the prevalence of MetShad increased significantly with increasing age (in studies in the Americas) (*p* = 0.006) (Figure 3). Also, the prevalence of MetS over time in studies in Asia was significantly increased (*p* = 0.024). Also, publication bias was not significant in the analyzed studies (*p* = 0.569).

## Discussion

The results of this study showed that one third of patients with RA have MetS. The results of a previous meta-analysis of 38 articles (with 70 groups) between 2007 and 2016 showed that the prevalence of MetS in patients with RA was 30.65%, which is almost consistent with the results of the present study (71). The reason for the high prevalence of metabolic syndrome in these patients can be attributed to traditional risk factors such as smoking, body mass index, gender, dyslipidemia and hypertension, although the role of continuous inflammation and activation of endothelial cells cannot be ignored (41). Inflammatory cytokines such as TNFα also reduce insulin function and facilitate insulin resistance (2). On the other hand, these patients use non-steroidal anti-inflammatory drugs (NSAIDs) and corticosteroids to control the disease, which can cause metabolic disorders such as high blood pressure, obesity and diabetes (27). Serum levels of some biomarkers associated with metabolic syndrome, adipokines such as adiponectin, and biomarkers of endothelial cell activation and inflammation may appear to be useful in predicting cardiovascular risk in patients with RA (72).

The highest prevalence of metabolic syndrome was related to studies in Asia and Europe and the lowest prevalence was related to studies in Africa. Given that nutritional, ethnic and sociodemographic status are the determinants of the prevalence of metabolic syndrome, the reason for this finding can be attributed to these differences in these communities.

In a study by Park et al. (73) the prevalence of metabolic syndrome in Korean and American adults was compared, and the results showed that the prevalence of metabolic syndrome and all its components (except low high density lipoprotein-cholesterol) was higher in American adults than in Korean. The two groups were not different in terms of blood pressure (73). The results of our study differ from those of Park et al. (73); in that they examined the prevalence of metabolic syndrome among patients with rheumatoid arthritis, not the general population. Therefore, further studies in this regard seem necessary.

The highest and lowest prevalence of metabolic syndrome were related to ATP III and EGIR criteria, respectively. In all diagnostic criteria, blood pressure, triglycerides, HDL cholesterol and fasting glucose are measured, and the difference between them is in the selection of the cut-off points and the measure of obesity. In WHO and EGIR criteria, the presence of hyperinsulinemia as an indicator of insulin resistance is the starting point, while in ATP III, the number of abnormalities is considered (69). These differences have led to different prevalence being reported in a group of patients (same patients) based on different criteria, so appropriate standards should be used to diagnose MetS in different regions. In a meta-analysis performed to estimate the prevalence of metabolic syndrome in postmenopausal women, the highest prevalence of metabolic syndrome was based on the ATP III screening criterion (74). The prevalence of metabolic syndrome increased significantly with age (in studies in the Americas). The prevalence of metabolic syndrome in the general population also increases with age (27), which can be due to redistribution of adipose tissue, weight gain, insulin resistance, and lipid changes (75).

Given that the prevalence of metabolic syndrome in patients with rheumatoid arthritis has not been studied in some countries and therefore has not been analyzed, the findings of this study should be generalized with caution worldwide.

## Conclusion

Metabolic syndrome is so common in patients with RA that one-third of these patients have MetS, so identifying at-risk patients is essential to prevent cardiovascular events.

## Data Availability Statement

The original contributions presented in the study are included in the article/supplementary material, further inquiries can be directed to the corresponding author.

## Author Contributions

WC: concept, design, and drafting of the manuscript. WC, MP, and XT: acquisition, analysis, or interpretation of data. XT: critical revision of the manuscript for important intellectual content. MP: statistical analysis. All authors gave their final approval of this version of the manuscript.

## Conflict of Interest

The authors declare that the research was conducted in the absence of any commercial or financial relationships that could be construed as a potential conflict of interest.

## Publisher's Note

All claims expressed in this article are solely those of the authors and do not necessarily represent those of their affiliated organizations, or those of the publisher, the editors and the reviewers. Any product that may be evaluated in this article, or claim that may be made by its manufacturer, is not guaranteed or endorsed by the publisher.
